# Impacts of Anthropogenic Pollutants on Benthic Prokaryotic Communities in Mediterranean Touristic Ports

**DOI:** 10.3389/fmicb.2020.01234

**Published:** 2020-06-09

**Authors:** Elena Tamburini, Lapo Doni, Raffaela Lussu, Federico Meloni, Giovanna Cappai, Alessandra Carucci, Enrico Casalone, Giorgio Mastromei, Francesco Vitali

**Affiliations:** ^1^Department of Biomedical Sciences, University of Cagliari, Cagliari, Italy; ^2^Department of Biology, University of Florence, Florence, Italy; ^3^Department of Civil-Environmental Engineering and Architecture, University of Cagliari, Cagliari, Italy; ^4^Institute of Agricultural Biology and Biotechnology, National Research Council, Pisa, Italy

**Keywords:** bacteria, archaea, next generation sequencing, metal, hydrocarbon, harbor, sediment, network

## Abstract

Ports and marinas are central nodes in transport network and play a strategic role in coastal development. They receive pollution from land-based sources, marine traffic and port infrastructures on one side and constitute a potential pollution source for the adjacent coastal areas on the other. The aim of the present study was to evaluate the effects of organic and inorganic co-contamination on the prokaryotic communities in sediments from three Mediterranean ports. The structure and composition of the bacterial and archaeal communities were assessed by targeted metagenomic analysis of the 16S rRNA gene, and the links of prokaryotic communities with environmental and pollution variables were investigated. The harbors presented pronounced site-specificity in the environmental properties and pollution status. Consistently, the structure of archaeal and bacterial communities in surface sediments exhibited a strong spatial variation among the three investigated ports. On the contrary, a wide overlap in composition of prokaryotic assemblages among sites was found, but local variation in the community composition and loss of prokaryotic diversity was highlighted in a heavily impacted port sector near a shipyard. We provided evidences that organic matter, metals and PAHs as well as temperature and salinity play a strong role in structuring benthic bacterial communities significantly contributing to the understanding of their responses to anthropogenic perturbations in marine coastal areas. Among metals, copper was recognized as strongly associated with the observed changes in bacterial assemblages. Overall, this study provides the first assessment of the effects exerted by multiple organic and inorganic contaminations on benthic prokaryotes in ports over a large spatial scale and designates bacterial community as a candidate tool for the monitoring of the sediment quality status in harbors.

## Introduction

Over the last two centuries, different manufactured materials and hazardous substances have been introduced in marine ecosystems by human activities causing their anthropization (Waters et al., 2016). The Mediterranean Sea is an interesting case study for investigating the impacts of anthropogenic pressures on marine ecosystems as it combines numerous maritime activities and demographic pressures (Piante et al., 2015). Moreover, its responsiveness to human pressures is accelerated by the oceanographic conditions of a semi-enclosed sea. The Mediterranean Sea is not only amongst the busiest routes of global maritime transport, but it is also the main tourism destination in the world, accounting for the 53% of EU passenger seaborne traffic in 2009 (Eurostat, 2011) and 30% of the total world tourists in 2012 (Plan Bleu, 2014). Therefore, the Basin is globally one of the main hotspots of vulnerability to pressures exerted by tourism activities. More specifically, marinas and recreational harbors are ubiquitous tourism infrastructures, with 940 marinas along the Mediterranean coasts in 2010 (Piante et al., 2015).

Ports and marinas are central nodes in the transport network and play a strategic role in coastal development. They receive pollution from land-based sources, marine traffic and port infrastructures on one side and constitute a potential pollution source for the neighboring coastal areas on the other. Port pollution may result from ship accidents, deliberate operational discharges from ships, land activities, ship bunkering, garbage, dust, dredging, port maintenance, ship air emission, sewage, and others (Vandermeulen, 1996). In the last decades, the negative effects on natural ecosystems caused by anthropogenic activities have received increasing attention by the EU environmental policies (Borja et al., 2010). In this context, port sustainability has become crucial for protection of coastal water quality, wildlife, and human health in port city destinations (Di Vaio and Varriale, 2018). On the other hand, port facilities are types of activities that can result in a water body designated as a Heavily Modified Water Body (HMWB, Water Framework Directive 2000/60/EC, WFD). Human activities to support specific uses (e.g., navigation) have indeed caused physical alterations (i.e., dredging, confinement) in the water body (i.e., port) basically modifying its hydromorphological properties (Ondiviela et al., 2012, 2013). The peculiar features of HMWBs justify the development of monitoring and remediation programs specific and adequate to port characteristics.

Among chemical contaminants, metals and polycyclic aromatic hydrocarbons (PAHs) are almost ubiquitous in anthropized coastal areas, especially in harbors, which usually exhibit higher concentrations than the adjacent zones (Angelidis and Aloupi, 1995; Merhaby et al., 2015; Schintu et al., 2015; Zakhama-Sraieb et al., 2016). The distribution of metals within the aquatic environment is controlled by complex processes of material exchange, which are altered by natural and anthropogenic factors (Christophoridis et al., 2009). Metals are natural components of metalliferous minerals, which are geographically distributed in a heterogeneous way. Therefore, the background values can vary widely in different geographic regions, even in non-anthropized environments depending on the abundance of such metalliferous minerals (Gadd, 2010). Anthropogenic activities alter the biogeochemical cycle by increasing the concentration of metals with respect to their natural background and modifying their speciation in the environment (UNEP, 2013). On the other hand, PAHs are derived from crude oil products (i.e., petrogenic PAHs) and incomplete combustion of organic matter (i.e., pyrolytic PAHs). Natural sources have been found to be marginal, while anthropogenic activities are generally considered to be the major source of PAHs into the marine environment (Baumard et al., 1998). Long-range aeolian PAH transport of fine combustion particles appears to dominate the oceanic PAH flux (Gustafsson et al., 1997). In ports and marinas, the predominance of pyrogenic emission sources has extensively been reported with marked differences in pollutant compositions among different sites and the coexistence of petroleum and pyrogenic PAHs in multi-sectoral harbors (McCready et al., 2000; De Luca et al., 2004; Sprovieri et al., 2007; Merhaby et al., 2015; Schintu et al., 2015; Vitali et al., 2019). Lastly, metals and PAHs entering the marine environments accumulate in sediments, which act as a long-term contaminant sink (Christophoridis et al., 2009). Sediments in anthropized coastal zones are therefore contaminated by complex mixtures of organic and inorganic pollutants exhibiting a range of multifaceted interactions with bacterial communities (Liu et al., 2017).

Bacteria play a pivotal role in determining the fate and distribution of contaminants in marine sediments by controlling the global PAH fluxes by degradation (Duran and Cravo-Laureau, 2016) and altering metal speciation (Gadd, 2010). More specifically, interactions with microorganisms can lead to either an increase (i.e., siderophore production, redox mobilization, acidification) or a decrease (i.e., exopolymer production, intracellular sequestration, redox immobilization, biomineral formation) in metal bioavailability (Gadd, 2010). On the other hand, essential and non-essential elements above threshold concentrations exert toxic effects on bacteria by different mechanisms, such as oxidative stress caused by reactive oxygen species (ROS, Lemire et al., 2013). The compounds with two or three aromatic rings (i.e., low-molecular-weight PAHs) are acutely toxic while those having four or more rings (i.e., high-molecular-weight PAHs) are generally genotoxic (Ghosal et al., 2016). Nevertheless, the simultaneous exposure to PAHs and metals result in more complex impacts than those exerted by the single pollutant due to additive, synergistic or antagonistic effects. Indeed, metals can affect PAHs degradation by changing the surface properties of bacterial cells and interfering with enzymes on one side (Biswas et al., 2015); on the other, degradation of PAHs by the cytochrome P450 generate ROS reducing the tolerance to toxic metals (Kuang et al., 2013). In marine ecosystems, the impact of pollutants on benthic communities may also depend on the system attributes, such as hydrology, tidal energy, and climatic conditions (Nogales et al., 2011). In the last decade, the effects of co-contamination by PAHs and metals on benthic prokaryotic assemblages in marine sediments have been addressed in few studies (Iannelli et al., 2012; Sun et al., 2012; Chiellini et al., 2013; Misson et al., 2016) and even less have pursued this challenging goal by exploiting NGS techniques (Sun et al., 2013; Quero et al., 2015).

With these premises, the general objective of this work was to evaluate the impacts of organic and inorganic co-contamination on the prokaryotic communities in port sediments. In the framework of the ENPI CBCMED project MAPMED, sediments were collected from three touristic ports located along the Mediterranean Sea. The pollution status of the three harbors has been recently determined by Chatzinikolaou et al. (2018) and Vitali et al. (2019). Moreover, their environmental properties have been defined based on the combined assessment of physical parameters, chemical variables (i.e., nutrients, pigments), and macrobenthic diversity (Chatzinikolaou et al., 2018). In this study, the structure and composition of the bacterial and archaeal communities were assessed in surface sediments from the three ports by targeted metagenomic analysis of the 16S rRNA gene, and the links between prokaryotic communities and both environmental and pollution variables were investigated.

## Materials and Methods

### Study Sites and Sampling

**FIGURE 1:**
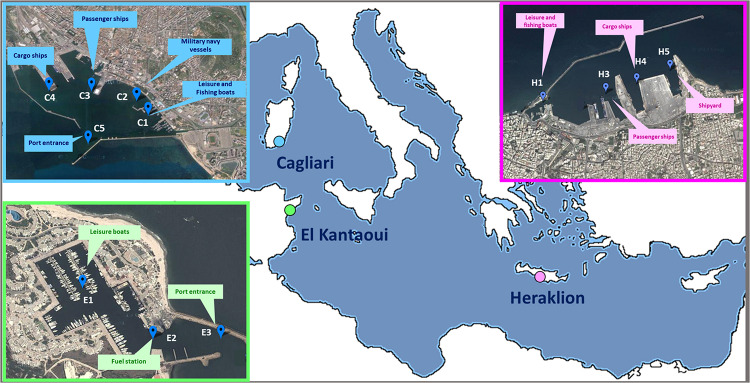
Maps of the touristic ports of Cagliari (C), El Kantaoui (E), Heraklion (H). The position of the sampling stations and the main sector uses are indicated on the maps. Map image: Google Earth Pro, Maxar Technologies.

A coordinated sampling campaign was performed in September 2012 at the end of the tourist season at three Mediterranean ports (Figure 1): Cagliari (Sardinia, Italy), El Kantaoui (Sousse, Tunisia), and Heraklion (Crete, Greece). Within each harbor, three to five sampling stations were located in sectors dominated by different port activities achieving an adequate spatial coverage of the whole port area (Supplementary Table S1). The collected samples were labeled as follows: the letter specifies the port (C: Cagliari; E: El Kantaoui; H: Heraklion), while the digit identifies the sampling station within each port sector (Supplementary Table S1).

The environmental parameters (Chatzinikolaou et al., 2018) included in the statistical analysis were salinity measured in surface seawater (S_W), temperature (T_S), redox potential (Eh), silt-clay ratio (SC), and organic carbon (OC). The sum of chlorophyll-a and phaeopigment concentrations (CPE, Danovaro et al., 1999) and the ratio of phaeopigments to the sum of chlorophyll-a and phaeopigments in sediments (PAP, Boon et al., 1998) were also calculated.

Concentrations of 31 individual aliphatic hydrocarbons (AHs) in the range C10-C40, Unresolved Complex Mixture (UCM), and 16 US EPA priority PAHs in superficial sediments were previously evaluated by gas chromatography-mass spectrometry in Chatzinikolaou et al. (2018) and Vitali et al. (2019). Abbreviations for the 16-EPA priority PAHs are as follows: 2-ring PAH – naphthalene (Naph); 3-ring PAHs – acenaphthylene (Aceph), acenaphthene (Ace), fluorene (Fl), phenanthrene (Phen), anthracene (Ant); 4-ring PAHs – fluoranthene (Flu), pyrene (Pyr), benzo[a]anthracene (BaA), chrysene (Chr); 5- ring PAHs – benzo[b]fluoranthene (BbF), benzo[k]fluoranthene (BkF), benzo[a]pyrene (BaP), indeno[1,2,3-c,d]pyrene (Inp), benzo[g,h,i]perylene (BgP), dibenzo[a,h]anthracene (DBA). The following hydrocarbon pollution descriptors were used: (i) the total level of AHs, calculated as the sum of individual compounds in the range C10-C40, (ii) the sum of four low-molecular-weight PAHs (Phen, Ant, Flu, Pyr, LPAHs) and the sum of eight high-molecular-weight (BaA, Chr, BbF, BkF, BaP, Inp, BgP, DBA, HPAHs) as descriptors of PAH pollution levels; (iii) the molar ratios of selected PAHs (ΣLPAH/ΣHPAH, Ant/Ant + Phen, Flu/Flu + Pyr, BaA/BaA + Chr, Inp/Inp + BgP), as descriptors of PAH sources (Vitali et al., 2019).

The concentrations of metals (Al, Cd, Cr, Cu, Fe, Ni, Pb, V, and Zn) and metalloids (As, Sb) were previously determined by Inductively Coupled Plasma optical emission spectrometry in Chatzinikolaou et al. (2018). A normalization of metal data by Al as conservative metal was applied according to the geochemical approach implemented for port sediments by Ho et al. (2012). In literature, Al has been extensively used as normalizer since it complies with a number of prerequisites (Schropp and Windom, 1988; Huntingford and Turner, 2011; UNEP/Med, 2011). More specifically, the element is one of the most important constituents of the aluminosilicate minerals, which represent the main group of minerals in the fine sediment fractions and tightly bind naturally occurring metals within their structure. Aluminum is also stable and is not significantly subject to environmental influences, such as reduction/oxidation, adsorption/desorption, and other diagenetic processes. Finally, the aluminum content is not generally influenced by anthropogenic sources.

### Analysis of Prokaryotic Communities by NGS of 16S rRNA Gene

Sample collection and DNA extraction were performed as previously described by Vitali et al. (2019). Briefly, three samples of surface sediments (0–1 cm) were collected using small plastic corers from each station (Chatzinikolaou et al., 2018). Extracted DNA (10 ng) was used as template in PCR reactions. The V4 region of the bacterial 16S rRNA gene was amplified by the bacterial-specific primer pair 563f/802r according to Claesson et al. (2010). For amplification of the archaeal 16S rRNA gene, a nested PCR approach was adopted. The first amplification was performed with archaeal-specific primers 21f/958r according to DeLong (1992). Then, the hypervariable V3 region of the 16S rRNA was amplified using the archaeal-specific primer pair 344f/519r (Yu et al., 2008). For each sample, three replicate reactions with each primer pair were combined to minimize stochastic PCR bias. The PCR products were purified from the agarose gel using the QIAquick Gel extraction kit (Qiagen). Sequencing was performed by the sequencing facility Source Bioscience (Nottingham, United Kingdom).

For data processing, raw sequences obtained by Illumina Miseq were demultiplexed by the sequencing facility. For pre-treatment, reads were quality checked with FastQC (Andrews, 2010), primers were removed with Cutadapt (Martin, 2011), and forward and reverse reads were merged using Pear (Zhang et al., 2014). The quality check with FastQC revealed a region of low quality at the end of the sequences. Thus, the sequences were subjected to a trimming and filtering step using Sickle (Joshi and Fass, 2011) and FastX-trimmer (Gordon and Hannon, 2012). A final quality control was carried out with MultiQC (Ewels et al., 2016) to evaluate the overall quality of the reads by aggregating the whole dataset. “Good Quality Reads” were subsequently imported into Quantitative Insights into Microbial Ecology (QIIME 2) version 2018-11 (Bolyen et al., 2019) and dereplicated. Illumina sequencing reads are available at the European Nucleotide Archive under accession study PRJEB36504.

For each distinct community (Bacteria, Archaea), the operational taxonomic units (OTUs) were assigned with a default identity of 97% using open reference OTU picking approach, then low abundance OTU < 0.005% (Bokulich et al., 2013), chimeras and singletons were identified and removed from the dataset, thus obtaining a filtered OTU-abundance table.

For each OTU, a representative sequence was used for taxonomy assignment against the Silva database release 132 (Quast et al., 2013). For the analysis of sulfate reducing bacteria (SRB), the OTUs assigned to the families Thermodesulfovibrionaceae, Desulfarculaceae, Dethiosulfovibrionaceae, Desulfobacteraceae, Syntrophaceae, and Syntrophobacteraceae were extracted from the normalized OTU-abundance table of Bacteria according to Robador et al. (2016). For community composition, the barplots and Venn diagrams were plotted using the ggplot2 package and the online tool Venny 2.1, respectively (Oliveros, 2015; Wickham, 2016).

### Statistical Analyses

Statistical analyses were performed using R (R Core Team, 2013) in RStudio (RStudio Team, 2015). Linear correlations between abiotic variables were computed by using the *corr.test* function of the psych package (Revelle, 2018) (Supplementary Figure S1). As pre-treatment transformation, data for each variable were subjected to the z-score transformation by subtracting their mean to each value and then dividing by their standard deviation. For multivariate analysis, the Principal Component Analysis (PCA) was performed on normalized (z-score) variables. PCA was obtained using *prcomp* function, while the *fviz_pca* function in factoextra package was used for plotting (Kassambara and Mundt, 2017). According to Ho et al. (2012), metal concentrations were included without normalization to Al in correlation analysis and PCA, while normalization was applied in all the other statistical tests.

In distance-based methods (i.e., Permutational multivariate analysis of variance, BIOENV, Mantel and partial Mantel tests), matrices were calculated by means of Bray–Curtis dissimilarity coefficient between sampling stations based on biotic data (i.e. Bacteria, Archaea, and SRB) by using the *vegdist* function in vegan R package (Oksanen et al., 2019), while Euclidean distance was calculated using the *dist* function based on abiotic data and geographical coordinates of the sampling stations.

For the two distinct prokaryotic communities (Bacteria, Archaea) read count data were firstly normalized by Cumulative Sum Scaling (CSS) transformation, using metagenomeSeq package (Paulson et al., 2013; Paulson, 2014). The indices of diversity (richness as number of observed OTU, Shannon with an e log base) and evenness (Pielou’s) were used to assess the alpha-diversity. All indices were calculated for all samples using the function *global* in the microbiome package (Lahti et al., 2017). Beta diversity was inspected by ordination analysis [principal coordinate analysis (PCoA)] based on Bray–Curtis dissimilarity using the function *ordinate* of the phyloseq package (McMurdie and Holmes, 2013). Permutational multivariate analysis of variance (PERMANOVA) was then used to evaluate the null hypothesis that there were no significant differences between ports. PERMANOVA was performed using the *adonis* function in the vegan package on the Bray–Curtis dissimilarity matrix with 9,999 permutations.

The relation between the structure of the prokaryotic communities (i.e., Bacteria, Archaea, SRB) and the measured abiotic variables was investigated by the BIOENV test. In order to identify the best subsets of variables associated with each community structure (BestBIOENV), the Spearman rank correlation coefficient between the matrix for abiotic variables (calculated with Euclidean distance) and the matrix of each distinct community (calculated with Bray–Curtis) was determined by the *bioenv* function in the vegan package. A Mantel test was then performed to assess the significance of the biotic-abiotic relation using the *mantel* function (9,999 permutations) in the vegan package.

The Mantel test was also applied to correlate each Bray–Curtis distance matrix of community structure with the Euclidean distance matrix of the best subset of abiotic variables (BestBIOENV) selected for each distinct prokaryotic community. The partial Mantel test was subsequently performed according to Vitali et al. (2019) to evaluate the relationship between the community structures and the best subsets of variables (BestBIOENV) after the effects of spatial autocorrelation have been removed. Briefly, the *mantel.partial* function in vegan package was used to determine the correlation between each Bray–Curtis distance matrix of community structure and the Euclidean distance matrix of the best subsets of variables (BestBIOENV) while controlling the effect of spatial autocorrelation with the Euclidean distance matrix of geographical coordinates of sampling stations (GEO).

A redundancy analysis (RDA) was performed on the Hellinger transformed OTU-abundance table (Legendre and Gallagher, 2001) to investigate the effects of the best subset of abiotic variables selected for each distinct prokaryotic community. The *rda* function in the vegan R package was used to test the independent and combined effects of the variables in the best subsets (i.e., model was formalized in R with the ^∗^ operator). The ANOVA test for constrained analysis was performed to assess the significance of the RDA model (i.e., overall and by terms) using the *anova.cca* function of vegan package.

To further investigate the relations between community structure and measured abiotic variables, Spearman correlation analysis was performed between all OTUs and between all OTUs and abiotic variables. Correlations were calculated with the *corr.test* function in the psych package, with false discovery rate correction for multiple testing (i.e., option “fdr” in *corr.test* function). For archaeal communities, few correlations were retained and therefore the dataset was not further analyzed. For bacterial communities, the strong and significant (i.e., spearman’s rho > 0.8 and *p*-val < 0.05) correlations were selected, which were imported in Cytoscape (Shannon et al., 2003) to construct and visualize a correlation network. In the network, each node is a genus-level OTU or an abiotic variable, and edge connecting two nodes indicate the presence of a significant and strong correlation between two nodes (i.e., between two bacteria genus or between a bacterial genus and an abiotic variable). Clusters in the network were identified and calculated with clusterMaker2 (Morris et al., 2011) using the Community Clustering (GLay) algorithm. Network properties were calculated with Cytoscape “NetwrokAnalyzer” plugin (assuming an un-directed network on the unclustered network) and node degree was used to color nodes in the network clusters.

## Results

### Environmental and Pollution Status

**FIGURE 2:**
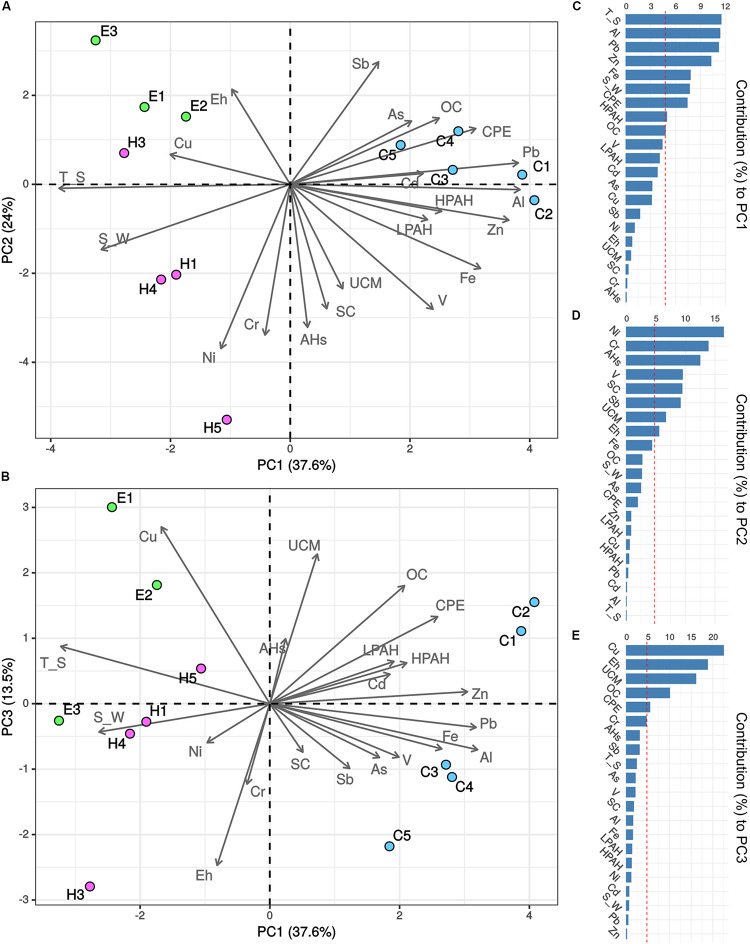
Principal component analysis (PCA) of environmental and pollution variables recorded in the ports of Cagliari (C, blue), El Kantaoui (E, green), and Heraklion (H, pink). Biplots showing **(A)** PC1 vs. PC2, **(B)** PC1 vs. PC3. The percentage of the variation explained by each axis is indicated in parentheses after the axis label. Variable contribution to the first **(C)**, second **(D)**, and third **(E)** component is reported as percentage.

A PCA analysis was performed including all the abiotic variables in order to identify which of them contributed the most to the description of the environmental and contamination status of the three investigated ports (Figures 2A,B). The first three components accounted for 75.1% of the total variance. Sediments collected in Cagliari were separated from those collected in the other two ports on the first component (PC1) based on higher concentrations of the four metals Al, Pb, Zn and Fe, higher levels of CPE as well as lower values of temperature and salinity (see vectors in Figures 2A,B, and variable contribution in Figures 2C–E). On the second component (PC2), higher levels of the three metals Ni, Cr, and V, aliphatic hydrocarbons (AHs, UCM) and silt-clay ratio as well as lower values of Sb separated the majority of Heraklion samples from sediments collected in El Kantaoui. Cagliari exhibited intermediate levels of the variables included in PC2. Finally, sediments collected from the inner part of the El Kantaoui port (E1, E2), in the sectors hosting the leisure boat (C1) and military navy vessels (C2) in Cagliari, and near the shipyard in Heraklion (H5) were separated from the other samples on the PC3 based on lower redox potential and higher levels of Cu, OC and UCM.

### Structure and Composition of Prokaryotic Communities

The richness displayed a low variation (coefficient of variation < 5%) with average values of 1,880 OTUs and 597 OTUs for Bacteria and Archaea, respectively. The only exception was the sediments collected near the shipyard in Heraklion (H5), which differed from all the other stations with 654 OTUs for Bacteria and 361 OTUs for Archaea. In station H5, the lowest values of Shannon (H′) and Pielou’s evenness (J’) were also found for both prokaryotic communities (Supplementary Table S2).

**FIGURE 3:**
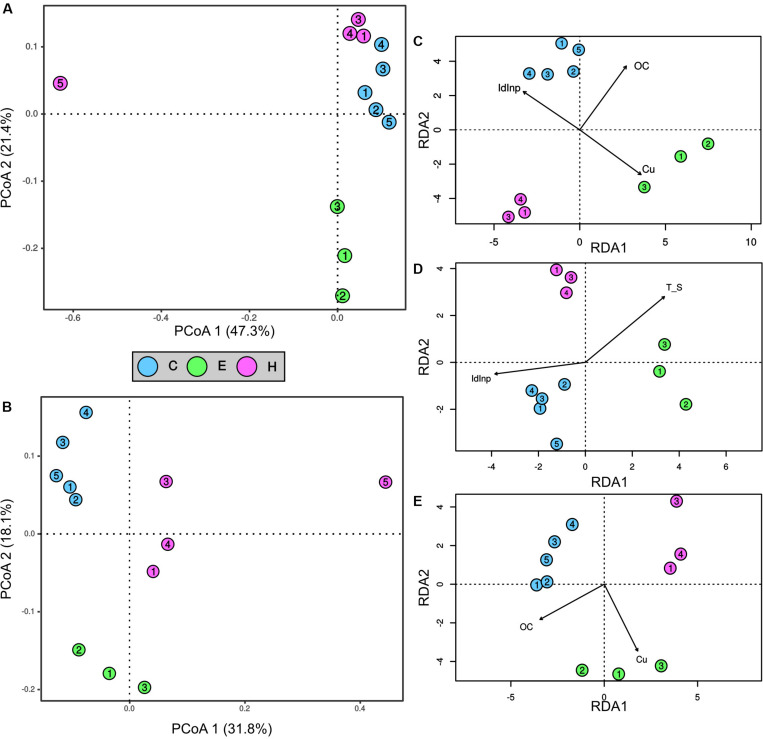
Ordination and contribution of abiotic variables to spatial variation of prokaryotic community structures in surface sediments collected at the port of Cagliari (blue), El Kantaoui (green), Heraklion (pink). PCoA ordination of bacterial **(A)** and archaeal **(B)** communities. The percentage of the spatial variation in community structure explained by each axis is indicated in parentheses after the axis label. Redundancy analysis (RDA) ordination diagrams of the first two axes for bacterial **(C)**, SRB **(D)**, and archaeal **(E)** communities. The constrained sets of environmental variables analyzed are indicated as vectors. Only significant constrained variables are reported.

The first two ordination axis of the PCoA analysis explained 68.7 and 49.9% of the variance in the bacterial and archaeal communities, respectively (Figures 3A,B). A clear segregation of the two prokaryotic communities in the ordination space was evident on the basis of the factor “port.” Indeed, the PERMANOVA analysis demonstrated that the port was a factor significantly (*p* < 0.01) affecting the bacterial and archaeal communities. Most noticeably, sediments near the shipyard (H5) differed from all the other samples in community structure of both Bacteria and Archaea (Figures 3A,B).

**FIGURE 4:**
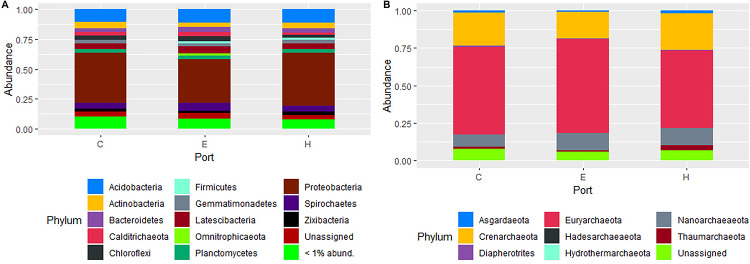
Composition of prokaryotic communities in surface sediments of the study ports (C: Cagliari, E: El Kantaoui, and H: Heraklion). Bar plot showing the average contribution for each port of Bacteria **(A)** and Archaea **(B)** at phylum level. Taxa that on average comprised less than 1% of the libraries were grouped. The station H5 was excluded from calculation of average values for the Heraklion port.

The most abundant phyla of Bacteria was Proteobacteria (41 ± 3.5%) followed by Acidobacteria (11 ± 0.8%), Latescibacteria (5.3 ± 0.8%), Spirochaetes (5.2 ± 0.9%), Actinobacteria (4.7 ± 0.8%), Bacteroidetes (3.5 ± 0.5%), Chloroflexi (3.3 ± 0.9%), Planctomycetes (3.2 ± 0.4%). For archaeal communities, the most abundant phyla were Euryarchaeota (58 ± 4.9%), followed by Crenarchaeota (21 ± 3.9%) and Nanoarchaeaeota (9.9 ± 2.5%). The other less abundant phyla were all below the 3%, while the unassigned sequences accounted on average for 2.1 ± 1.0 and 3.5 ± 2.5% in the composition of bacterial and archaeal communities, respectively. The community contribution of Bacteria and Archaea for each port is shown in Figure 4. Proteobacteria showed the highest values in Heraklion (44 ± 1.8%) and the lowest in El Kantaoui (36 ± 0.9%), with intermediate percentages in Cagliari (42 ± 1.7%). Sediments from El Kantaoui showed the highest percentages of Euryarchaeota (63%) and Heraklion the lowest one (51%), while Cagliari (58%) displayed intermediate values. A total of 2,482 bacterial OTUs and 909 archaeal OTUs were identified across all samples and, among them, 87 and 73% were shared among the three ports for Bacteria and Archaea, respectively (Supplementary Figure S2). Actually, sediments collected near the shipyard in Heraklion (H5) exhibited a peculiar community composition as compared to all the other samples (Supplementary Figure S2). More specifically, the highest percentages of Spirochaetes (12%), Actinobacteria (8.2%), and Firmicutes (7.8%) as well as the lowest percentages of Proteobacteria (26%), Acidobacteria (3.3%), Latescibacteria (3.3%), Chloroflexi (2.6%), and Planctomycetes (1.5%) were found in station H5, which also exhibited the highest percentages of Thaumarchaeota (11%) and Asgardaeota (3.0%). Because of its peculiar community compositions, sediments collected in station H5 were not included in all the average calculations of community composition (Figure 4) as well as in subsequent analyses.

### Relation Between Prokaryotic Communities and Environmental and Pollution Variables

The linking between the measured abiotic variables and the prokaryotic communities of Bacteria and Archaea was predicted using the BIOENV test. Moreover, the specific group of SRB was separately analyzed for its crucial role in ecosystem functioning in marine sediments. The best subsets of abiotic variables predicted by BIOENV test were: (i) concentrations of OC and Cu for Archaea (ρ = 0.9025, *p* = 0.0001), (ii) the concentrations of OC and Cu as well as a descriptor of PAH sources, namely the Inp/Inp + BgP ratio for Bacteria (ρ = 0.8060, *p* = 0.0001), (iii) temperature, OC, Cu, and the Inp/Inp + BgP ratio for SRB (ρ = 0.8400, *p* = 0.0002). The relation between each prokaryotic community and the best subset of abiotic variables predicted by BIOENV, was further explored by RDA ordination analysis (Figures 3C–E). The analysis confirmed significant relations between the community structure and each single abiotic variable predicted by BIOENV for Bacteria and Archaea (ANOVA, *p* < 0.05), but interactions between abiotic variables were never significant in ANOVA by term tests (ANOVA, *p* > 0.05). For SRB community, RDA showed a significant effect of the single variables temperature and the Inp/Inp + BgP ratio (*p* < 0.05), while the single variables OC and Cu, as well as the interactions among abiotic variables were not significant in ANOVA by term tests (ANOVA, *p* > 0.05).

Cogently the results of PERMANOVA analysis, Mantel test (Table 1) confirmed the presence of significant correlations (*p* < 0.05) between the geographic locations of the sampling stations and the community structures of Bacteria, Archaea, and SRB (spatial autocorrelation; row “GEO” in Table 1). Correlations were weak for SRB (ρ = 0.3450), moderate for Bacteria (ρ = 0.4794), while Archaea showed the highest correlation between community structure and geographic locations of the sampling stations (ρ = 0.6805). The effect of the best subsets of abiotic variables was thus untangled from the one owed to autocorrelation by partial Mantel test (Table 1, row “BestBIOENV – GEO”). The partial Mantel test demonstrated a significant and strong correlation (ρ > 0.8, *p* < 0.001) between each prokaryotic community and the best subset of selected abiotic variables (Table 1, row “BestBIOENV – GEO”).

**TABLE 1:** Spearman correlation coefficients (R) of Mantel and partial Mantel tests between the distance matrices of the best subsets (selected by the BIOENV test) of abiotic variables (Euclidean distance), geographical locations (Euclidean distance), and community structures (Bray–Curtis) of Bacteria, Archaea and SRB.

**Test**	**Matrices**	**Bacteria**	**SRB**	**Archaea**
Mantel	BestBIOENV	0.8463**	0.8438**	0.8929**
	GEO	0.4794*	0.3450^+^	0.6805**
Partial Mantel	BestBIOENV – GEO	0.8038**	0.8378**	0.8294**

*BestBIOENV: organic carbon, Cu (Al normalized), Inp/Inp + BgP ratio for Bacteria; organic carbon, Cu (Al normalized) for Archaea; temperature, organic carbon, Cu (Al normalized), Inp/Inp + BgP ratio for SRB. GEO: geographical coordinates of the sampling stations. – GEO: the influence of distance matrix GEO is removed. +*p* < 0.05, ^∗^*p* < 0.01; ^∗∗^*p* < 0.001.*

**FIGURE 5:**
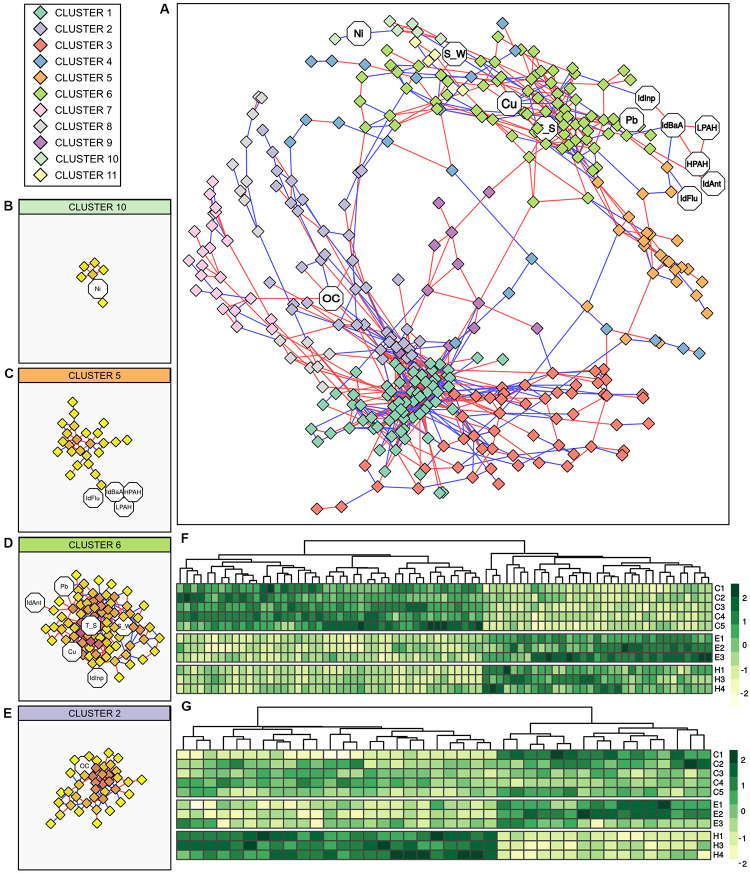
Correlation analysis between bacterial genus-level OTUs and abiotic variables. **(A)** Network constructed with strong and significant Spearman correlations between OTUs, and between OTUs and abiotic variable. Node represents OTUs (rhombi) or abiotic variables (octagons). Node color is based on results of network clusterization analysis. Edges represent strong and significant Spearman correlation between nodes. Edge color is based on the direction of the correlation (blue for negative correlations, red for positive correlations). **(B–E)** Detailed view of the identified clusters in the network comprising abiotic variables. In those detailed views, node color is based on node degree (i.e., the number of nodes connected to a node). **(F)** Heatmap showing the distribution of OTUs in cluster 6 between samples. **(G)** Heatmap showing the distribution of OTUs in cluster 2 between samples. IdAnt: Ant/Ant + Phen; IdBaA: BaA/BaA + Chr; IdFlu: Flu/Flu + Pyr, IdInp: Inp/Inp + BgP; LPAHs: Phen, Ant, Flu, Pyr; HPAHs: BaA, Chr, BbF, BkF, BaP, Inp, BgP, DBA, S_W: salinity in surface water; T_S: temperature in surface sediments.

The relationship between the bacterial communities and abiotic variables was further investigated by correlation analysis. Figure 5 reports results of Spearman correlation analysis of the bacterial communities (cumulating all samples collected from the three ports and excluding the station H5) as a correlation network. Constructed network was composed of a total of 32 elements, 434 nodes (17 abiotic variables, 417 genus-level OTUs), and 1,131 edges (500 negative correlations, 631 positive correlations). The main element accounting for the majority of nodes and edges was used for further analysis. This main element was composed of 355 nodes (12 abiotic variables, 343 genus-level OTUs) and 1,077 edges (484 negative correlations, 593 positive correlations), while the average number of nodes connected to each node (i.e., the average node degree) was 6.07 (*SD* = 5.95). The network was divided into 11 clusters based on node connectivity (Figure 5). Overall, two distinct modules of genus-level OTUs can be observed in the network topology/morphology (Figure 5A). Those modules were not generated by groups of mutual excluding OTUs, as positive (which could be interpreted as co-occurrence) and negative (which could be interpreted as mutual exclusion) edges were evenly distributed across the network. Moreover, inspection of the aforementioned modules showed that the OTUs distribution was not ascribable to differences in community composition among ports, a strong factor shaping the bacterial communities. The two modules were rather connected to different group of genera, having different relationships with abiotic variables. More specifically, the module on the lower part of the network was almost exclusively composed of genus-level OTUs and comprised only one abiotic variable, namely OC. The main clusters of this module were clusters 1, 2, 3, and 7. The clusters 1, 3, and 7 were exclusively composed of OTUs, cluster 1 showed the higher node degree values (data not shown), while cluster 2 comprised the abiotic variable OC (Figure 5E). Conversely, the module on the upper part of the network comprised all the connected abiotic variables. Main clusters of this module (Figures 5B–D) were cluster 6 (connected with the abiotic variables Ant/Ant + Phen, Cu, Inp/Inp + BgP, Pb, T_S, S_W), cluster 5 (connected with the abiotic variables BaA/BaA + Chr, Flu/Flu + Pyr, HPAHs, LPAHs) and cluster 10 (connected with the abiotic variable Ni).

The heatmaps showing the genus-level OTUs distribution among ports of cluster 6 (as the main abiotic connected cluster in the upper module) and cluster 2 (as the only abiotic connected cluster in the lower module) are shown in Figure 5. Even if the overall network did not reflect differences in genus-level OTUs distribution among ports, cluster 6 showed a marked similarity in OTUs composition and abundance distribution between El Kantaoui and Heraklion and a substantially different pattern in Cagliari. On the other hand, cluster 2 highlighted similarity between Cagliari and El Kantaoui, while Heraklion was substantially different. Mirroring abundance distribution, the PCoA ordinations of the OTUs included in cluster 2 and cluster 6 showed similar separations among the three ports with the first axis explaining 81.8 and 82.5% of the total variance, respectively (data not shown).

Upon detailed inspection of the OTUs included in cluster 2 (*n* = 40), four OTUs established a direct relationship with the abiotic variable OC. More specifically, an OTU assigned to the genus *Sedimenticola* exhibited a positive correlation with OC and a negative correlation with a second OTU belonging to the order Clostridiales, which in turn was negatively correlated to OC. Moreover, a positive correlation with OC was found for one OTU assigned to the uncultured lineage SJA-28 in the class Ignavibacteria and a negative correlation for an OTU attributed to the class Gammaproteobacteria. Among the OTUs included in cluster 6 (*n* = 78), the abiotic variables with the highest number of correlations were Cu (Node degree = 8) and sediment temperature (Node degree = 12), which established complex relationship with a total of 16 OTUs. Overall, those OTUs displayed a high connectivity level, with 5.5 median node degree (i.e., the median number of node connecter to each node of this network module) and values ranging from 1 to 12. Four OTUs established direct relationships with both Cu and temperature. Among them, two OTUs identified as belonging to the class Thermodesulfovibrionia and one to the phylum Schekmanbacteria were negatively correlated to Cu and temperature, while one OTU assigned to the class Anaerolineae was positively correlated to both variables. In addition to these four shared OTUs, four and eight OTUs were correlated singularly to Cu and temperature, respectively. More specifically, two OTUs were negatively correlated to Cu and assigned to the order Tistrellales in the class Alphaproteobacteria and to the uncultured clade BD7-8 in the class Gammaproteobacteria. On the other hand, Cu was positively correlated to one OTU identified as belonging to the genus *Sulfurovum* and one OTU assigned to the family Ruminococcaceae. As far as sediment temperature is concerned, negative correlations were found with one OTU assigned to Candidatus Moranbacteria order in the phylum Petescibacteria and one OTU assigned to the JS1 group in the phylum Atribacteria, while a positive correlation to temperature was found for six OTUs affiliated to the genus *Desulfosarcina*, the class Phycisphaerae, and the families Gemmatimonadetes, Spirochaetaceae, Oligoflexaceae, and Pedosphaeraceae.

Among the other abiotic variables included in cluster 6, one OTU identified as belonging to the Candidatus phylum Moranbacteria exhibited a negative correlation with salinity in surface water. A positive correlation with salinity was found for one OTU assigned to the family Terasakiellaceae and one OTU assigned to the class Gammaproteobacteria in the clade KI89A. The metal Pb correlated negatively to a single OTU identified as belonging to the genus Alkalispirochaeta and positively to two OTUs, one assigned to the family Nitrosococcaceae and one to the phylum Lentisphaerae. Finally, a single OTU affiliated to the family Desulfobacteraceae was negatively correlated to the Inp/Inp + BgP ratio and an unassigned OTU was positively correlated to the Ant/Ant + Phen ratio.

## Discussion

Prokaryotic communities play a fundamental role in ecosystem functioning in marine sediments regulating essential processes in global biogeochemical cycles, organic and inorganic contaminant transformation, and pollutant bioremediation. Recently, the impacts on benthic communities of organic and inorganic co-contamination as multiple stressors in harbors have been addressed by an increasing number of studies, such as extensive characterizations of complex commercial ports (Iannelli et al., 2012; Chiellini et al., 2013; Misson et al., 2016) as well as comparison between ports (as pollutant hot spots) and more natural (or less contaminated) coastal sediments (Sun et al., 2012, 2013). In this background, the present work represents the first assessment of the combined effects of multiple organic and inorganic pollutants on benthic prokaryotes in different ports at a large spatial scale (i.e., Mediterranean basin).

The sediment contaminations in the ports under study are markedly heterogeneous in compositions, levels and emission sources. Overall, the levels of PAHs vary over three orders of magnitude (25–49,000 ng/g), covering the range of concentrations previously reported for Mediterranean harbors (Vitali et al., 2019). The surface sediments in the artificial marina of El Kantaoui presents the highest levels of Cu (181 ± 10 mg/kg), a metal extensively used as antifouling agent in paints for ship hulls (Dahllöf and Andersen, 2009). Indeed, the copper level is three orders of magnitude higher inside the marina than in the adjacent coastal sediments (Zakhama-Sraieb et al., 2016). On the other hand, sediments in the El Kantaoui port are contaminated by low/moderate levels of PAHs with fuel combustion as primary emission source (Vitali et al., 2019). The Heraklion port is characterized by a moderate level of PAHs emitted by different sources. Moreover, the co-occurrence of Ni (345 ± 11 mg/kg) and Cr (98 ± 43 mg/kg) was found, which could be reasonably ascribed to anthropogenic sources, such as nickel-chrome plating (Dahllöf and Andersen, 2009). The port of Cagliari shows the highest levels of PAHs, primarily originated by burning of coal and biomass (Vitali et al., 2019). As far as metals are concerned, sediments exhibit a 10-fold higher level of Pb (156 ± 38 mg/kg) than the other studied sites. Notably, the Cagliari port is located in the context of a peculiar mineralogical background near an important abandoned mining district (Cidu and Fanfani, 2002), even if anthropogenic inputs could not be ruled out inside the port area (Schintu et al., 2016). Finally, the three studied ports are markedly different in terms of environmental properties because of their different geographical positions in the Mediterranean Sea and local factors (Chatzinikolaou et al., 2018).

As expected for such a pronounced site-specificity in abiotic conditions, the archaeal and bacterial communities exhibited a strong spatial variation among the three investigated ports (Figure 3). On the contrary, we found an unforeseen overlap in composition of prokaryotic communities (Figure 4), down to the lowest taxonomic rank (i.e., OTU level, Supplementary Figure S2). On this core of shared taxa, the benthic communities in sediments collected near the shipyard in Heraklion clearly moved away from all the other stations for their structure and composition as well as the lowest richness and evenness. More specifically, Firmicutes and Spirochaetes were six- and two-fold more represented as compared to the other studied sediments, respectively. An increase in the relative abundance of Firmicutes has been found under anoxic conditions as compared to oxic ones (McKew et al., 2013). On the other hand, Spirochaetes are common and abundant in anoxic contaminated sites, where they have been suggested to drive necromass recycling (Dong et al., 2018). It is worth noting that the three most abundant OTUs were affiliated to the phylum Aegiribacteria and the family Ruminococcaceae. Members of the phylum Aegiribacteria have been found in an extreme meromictic system under anoxic conditions (Hamilton et al., 2016), while Ruminococcaceae are well-known anaerobic bacteria. The fourth most abundant OTU belonged to the phylum Acetothermia (previously candidate OP1 phylum), which has been involved in biogeochemical transformations in oil reservoirs (Hu et al., 2016). The peculiar prokaryotic assemblage in the Heraklion shipyard station is paralleled by the distinct abiotic status of its sediments (Figure 2), which exhibit the finest particle size, the most strict anoxic conditions, and the highest contamination by aliphatic hydrocarbons, petrogenic PAHs, and Ni throughout all stations, but also the highest amount of organic carbon among Heraklion stations (Chatzinikolaou et al., 2018; Vitali et al., 2019). The strict anoxic condition and pollution status in Heraklion shipyard sediments can be reasonably ascribed to the high organic carbon load and consequent increased respiration (Acosta-González et al., 2015) as well as to the high contaminant sorption and the slow oxygen diffusion in fine grain size sediments (Eggleton and Thomas, 2004). Consistently with our results, a negative impact on diversity of benthic prokaryotic assemblages with a reduction of species richness and changes in community structure has been extensively documented in chronic contaminations by petrogenic hydrocarbons (Orcutt et al., 2010; Acosta-González et al., 2015). Notably, a coordinated study of the three investigated Mediterranean ports also found the most heavily disturbed conditions in the sediments near the shipyard in Heraklion, as highlighted by the lowest species richness of the macrozoobenthic communities (Chatzinikolaou et al., 2018) and a “poor” ecological status (“unacceptable” under the WFD) based on benthic macrofaunal indices (Dimitriou et al., 2020).

The different statistical approaches implemented in this study demonstrated a strong link between the prokaryotic communities and organic matter in port sediments with numerous connections among bacterial genus-level OTUs (Figure 5). More specifically, a first module in the network gathers bacterial genera, which mainly take part to complex biotic interactions and are basically unrelated to abiotic variables except for a small proportion of taxa interconnected with OC (Figure 3A, lower module). A direct positive link with OC was found in the network for Ignavibacteria and *Sedimenticola*. The cultivable members of Ignavibacteria are facultatively anaerobic with an obligately organotrophic mode of life either by fermentation or respiration with several electron acceptors (Podosokorskaya et al., 2013). On the other hand, *Sedimenticola* have been demonstrated to grow autotrophically by sulfur oxidation coupled to denitrification under hypoxic or anaerobic conditions, but also organotrophically under aerobic and anaerobic conditions (Flood et al., 2015). In line with these results a role in decomposition of organic materials in the investigated surface sediments may be suggested for these recently described groups characterized by a pronounced metabolic versatility. Overall, opposite trends between Cagliari and El Kantaoui on one side, and Heraklion, on the other, were found in genus-level OTUs distribution in cluster 2 (connected with the abiotic variable OC, Figure 5F), reasonably related with the highest amount of organic matter in sediments (Figure 2, PC3). Cogently, RDA assigned the detected variations in bacterial community structure to changes in organic carbon levels with opposite trends for Heraklion and the other two ports (Figure 3C). We found neither an interaction of OC with Cu or descriptors of PAH sources in RDA nor a direct interconnection with other abiotic variables in the network. Our results should not be interpreted as a lack of reciprocal effects between pollutants and organic matter. It is well-known that the organic carbon fraction in sediments plays an important role in binding metals and hydrophobic contaminants, including PAHs. However, organic matter is present in sediments in different forms that may have very different sorption capacities for hydrophobic contaminants. Therefore, the nature of organic matter (e.g., coal, vegetable debris) determines the bioavailability, biodegradability and biological effects exerted by PAHs in sediments (Yunker et al., 2002; Ghosh et al., 2003). On the other hand, the ability to bind toxic metals in the colloidal fraction of the organic carbon pool is important in the cycling of metals in aquatic systems (Ford and Ryan, 1995).

Network analysis identifies a second module composed of connections among bacterial taxa as well as strong relationships between them and abiotic parameters (Figure 5A, upper module). More specifically, there were numerous connections between OTUs affiliated to different lineages and temperature, and to a lesser extent with salinity. Both temperature and salinity have been found to be important drivers of bacterial communities over large spatial scale in coastal sediments (Sun et al., 2013; Bargiela et al., 2015). In the network, several OTUs ascribable to SRB (i.e., *Desulfosarcina* and Thermodesulfovibrionia) exhibited positive link with temperature. Accordingly, we also found a strong relation between this abiotic variable and the structure of SRB communities. These findings are consistent with previous results, which have demonstrated the prevailing ambient temperature exerts strong environmental selection on the composition of the SRB community in marine sediments from different climatic regions (Robador et al., 2016). Overall, temperature seems to play an important role in shaping SRB communities in sediments from the three investigated ports. In this context is important to mention that the Mediterranean Sea is characterized by well-known longitudinal gradients with a west to east increase in salinity and temperature (e.g., Coll et al., 2010), which may at least partially account for the correlation between the bacterial assemblages and the geographical locations of sampling stations. Moreover, the specific hydromorphological properties in ports (i.e., confinement) may reasonably contribute to local variation in salinity and temperature in the studied harbors, contributing to the site specificity of the benthic prokaryotic assemblages observed in this study. On the other hand, temperature has a crucial effect on the fate of PAHs and metals as it causes marked changes in the interactions (i.e., degradation, transformation, accumulation) between microorganisms and pollutants (Liu et al., 2017). Indeed, the solubility as well as the adsorption capacity and adsorption intensity on microbial cells and abiotic particles of PAHs and metals increase with increasing temperature (Liu et al., 2017). Even if descriptors of PAHs (i.e., Ant/Ant + Phen, BaA/BaA + Chr, Flu/Flu + Pyr, Inp/Inp + BgP, HPAHs) and metals (i.e., Cu, Pb, Ni) are connected with bacterial OTUs in the network, the biotic interconnections are more numerous (Figure 5A). Notably, the variable HPAHs interacts with a single OTU, while the variable LPAHs takes no direct connection with any genus-level OTU. This result is in line with that recently obtained for metals by Coclet et al. (2019) in a study on bacterioplankton in Toulon Bay; the authors have suggested that metals significantly influence the dynamics of few microbial groups, and could rather influence indirectly, via biotic interactions, the whole bacterial community. A clear separation of the Cagliari port from the other two ports was found based on the genus-level OTUs distribution in cluster 6. This result clearly mirrors differences in environmental and pollution status described by the abiotic variables included in this cluster (Figure 2, PC1).

Statistical analyses employed in this study cogently highlighted a strong link between descriptors of PAH sources and the structures of the whole bacterial communities as well as the group of SRB. These results support our previous study, which has recently provided a first evidence of the role of PAH emission sources in structuring the benthic communities of SRB as targeted by terminal restriction fragment length polymorphism of the *dsrAB* (dissimilatory sulfite reductase) gene (Vitali et al., 2019). Indeed, the combustion process by which PAHs are formed determines not only the composition of the contaminant mixture but also bioavailability of PAHs in sediments (Akkanen et al., 2012). In the network, we found a negative correlation between diagnostic ratio of PAH emission sources and Desulfobacteraceae. Bacteria belonging to Desulfobacteraceae have been previously found to be the dominant microorganisms in anaerobic enrichment cultures able to oxidize phenanthrene under sulfate reducing condition (Davidova et al., 2007; Himmelberg et al., 2018). Collectively, these findings might suggest the relative abundance of Desulfobacteraceae decrease in sediments where burning of biomass combustion was the dominant pollution source due to the low bioavailability of PAHs.

Among the investigated metals, both bacterial and archaeal communities showed a strong link with copper contamination in surface sediments. Our observations comply with similar studies on metal pollution in coastal areas, where copper has been suggested to be responsible for driving community changes in bacterioplankton from Toulon Bay (Coclet et al., 2019) and benthic bacteria from Australian coastal sediments (Sun et al., 2012). Moreover, a coordinated study on the three Mediterranean harbors investigated in the present work has previously demonstrated that copper affects macrobenthonic assemblages (Chatzinikolaou et al., 2018), a well-consolidated ecological tool for sediment quality assessment (McPherson et al., 2008; Gislason et al., 2017). The network analysis employed in this study allows us to identify potential copper sensitive- and tolerant- OTUs in the benthic bacterial community. More specifically, OTUs assigned to uncultured lineages of Alphaproteobacteria, Gammaproteobacteria, Schekmanbacteria, and Thermodesulfovibrionia seem to be negatively impacted by copper. In literature, similar results can be documented for Alphaproteobacteria and Gammaproteobacteria (Yin et al., 2015). Currently, Thermodesulfovibrionia are known for reduction of sulfate and other sulfur compounds but this class has not been previously linked to metals. The biological and geochemical importance of Candidatus phylum Schekmanbacteria is still unclear. On the other hand, the relative abundance of *Sulfurovum*, Anaerolineae and Ruminococcaceae increases with higher copper levels in the investigated sediments. Consistently with our results, an increase has been found in literature in metal contaminated environments including mangrove sediments and hydrothermal vents for Sulfurovum (Nakagawa et al., 2007; Fernández-Cadena et al., 2020), river sediments, soils, and coastal sediments for Anaerolineae (Sun et al., 2013; Yin et al., 2015; Meng et al., 2019), as well as barrier for mine tailings for Ruminococcaceae (Zhang et al., 2019).

## Conclusion

Our study gives strong evidences supporting the notion that organic matter, metals and PAHs as well as temperature and salinity shape prokaryotic communities in port sediments. The “port” is the main factor affecting the structure of archaeal and bacterial communities in surface sediments, a result consistent with the pronounced differences in sediment pollution status, geological background, and geographical position among the investigated sites. Nevertheless, a marked overlap in the composition of prokaryotic communities was found among ports. In this contest, the targeted NGS analysis of the benthic bacterial community allows us to detect local variation in the community composition and loss of prokaryotic diversity in the heavily impacted sediments near the shipyard in Heraklion. On the other hand, multiple statistical tools recognize copper as strongly associated with the observed changes in structure and composition of the benthic bacterial community and allows us to identify the bacterial populations more directly linked to the pollutant. These findings would deserve further investigations under controlled experimental conditions to verify a direct causal relation between stressor and candidate indicators and a more in-depth analysis of bacterial genomes and functions. Overall, the results obtained under the umbrella of the ENPI CBCMED project MAPMED designate the benthic bacterial community as a good candidate tool for monitoring of the sediment status in port management, a crucial prerequisite to plan bioremediation intervention, and lay the foundation for the developing of a proper benthic microbiota-based index of sediment quality status. In a wider perspective, our results provide a significant contribution to the understanding of responses of benthic prokaryotic communities to anthropogenic perturbations in marine coastal areas. Admittedly, the main limitation of the employed DNA-based target metagenomic analysis resides in the substantial inability to directly evaluate functional and metabolic pathways in the active community, and how bacteria are affected by and affect anthropogenic pollutants, a goal that will be pursued by untargeted metagenomic analysis coupled with transcriptomics.

## Data Availability Statement

The datasets generated for this study can be found in the https://www.ebi.ac.uk/ena/data/view/PRJEB36504.

## Author Contributions

ET, AC, and FV developed the concept for this study. ET and GC carried out the field work. ET, AC, GM, and EC designed the experiments and contributed reagents, materials, and analysis tools. FV and ET performed the microbiological experiments. GC performed the analysis of metals and metalloids. LD, FM, and ET analyzed abiotic data. FV and LD performed bioinformatic analyses. ET, LD, FV, and RL drafted the manuscript. All authors interpreted the results, edited and reviewed the final version of the manuscript and approved it before submission.

## Conflict of Interest

The authors declare that the research was conducted in the absence of any commercial or financial relationships that could be construed as a potential conflict of interest.
