# Supplementary material for: Impacts of Anthropogenic Pollutants on Benthic Prokaryotic Communities in Mediterranean Touristic Ports
Source: Front Microbiol. 2020 Jun 9;11:1234. doi: 10.3389/fmicb.2020.01234 (PMC7326019; doi:10.3389/fmicb.2020.01234)

Figure S1. Linear correlation between environmental and pollution variables recorded in the ports of Cagliari, El Kantaoui, and Heraklion. Not significant correlations (p>0.05) are blank, while significant correlations (p<0.05) are colored in a scale from blue colors for positive correlations to red colors for negative correlations (see legend).

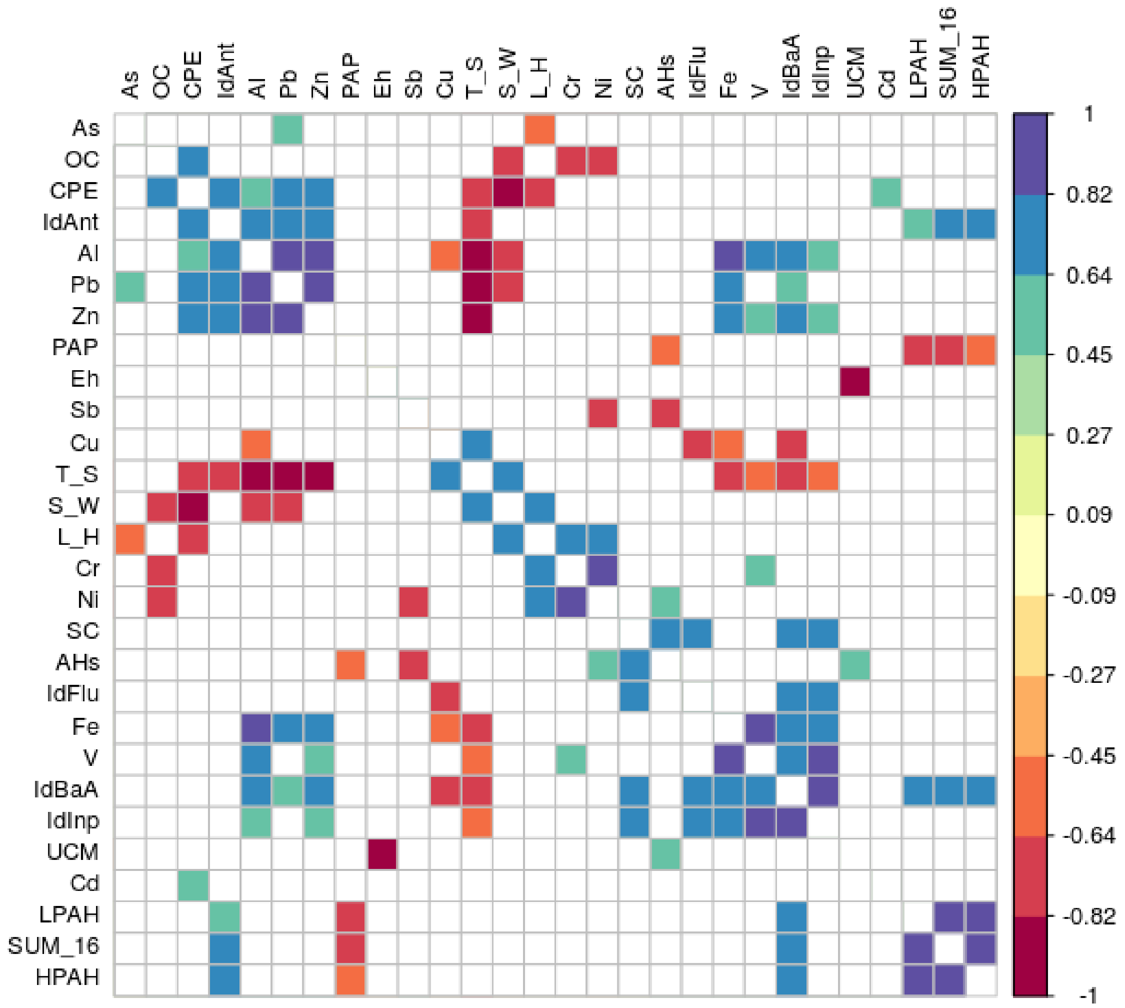

Supplement: Supplementary file 1 [file Image_1.pdf]
