# Supplementary material for: Impacts of Anthropogenic Pollutants on Benthic Prokaryotic Communities in Mediterranean Touristic Ports
Source: Front Microbiol. 2020 Jun 9;11:1234. doi: 10.3389/fmicb.2020.01234 (PMC7326019; doi:10.3389/fmicb.2020.01234)

Figure S2. Composition of prokaryotic communities in surface sediments collected at each sampling station in the study ports (C: Cagliari, E: El Kantaoui, and H: Heraklion). Bar plot showing the contribution of Bacteria **(A)** and Archaea **(B)** at phylum level. Venn chart showing the overlap among ports of bacterial OTUs **(C)** and archaeal OTUs **(D)**. Cagliari (blue), El Kantaoui (green), and Heraklion (pink, station H5 was excluded).

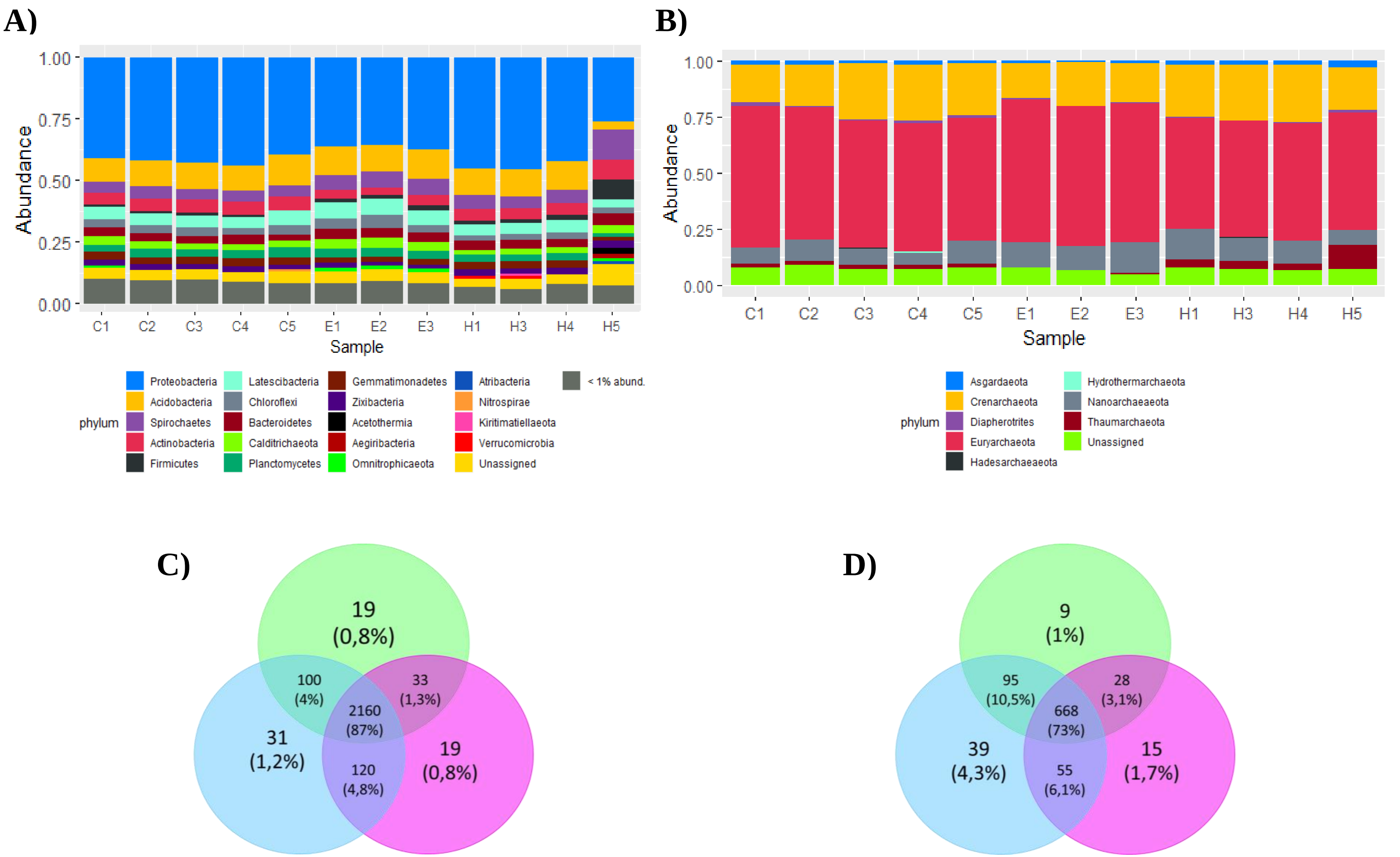

Supplement: Supplementary file 2 [file Image_2.pdf]
