# Supplementary material for: Impacts of Anthropogenic Pollutants on Benthic Prokaryotic Communities in Mediterranean Touristic Ports
Source: Front Microbiol. 2020 Jun 9;11:1234. doi: 10.3389/fmicb.2020.01234 (PMC7326019; doi:10.3389/fmicb.2020.01234)
Supplement: Supplementary file 3 [file Table_1.docx]

**Table S1.** Mediterranean touristic ports selected as the study sites (Vitali et al., 2019).

| **Port** | **Water surface**  **(km^2^)** | **Station label** | **Depth** | **Longitude** | **Latitude** | **Sector** **use** |
| --- | --- | --- | --- | --- | --- | --- |
| Cagliari  (Sardinia, Italy) | 2.07 | C1 | 7.8 | 39.203163 | 9.123244 | Leisure and fishing boats |
|  |  | C2 | 4.5 | 39.205534 | 9.121158 | Military navy vessels |
|  |  | C3 | 8.3 | 39.207213 | 9.112875 | Passenger ships |
|  |  | C4 | 13.5 | 39.207200 | 9.105139 | Cargo ships |
|  |  | C5 | 11.4 | 39.198729 | 9.112279 | Port entrance |
| El Kantaoui  (Tunisia) | 0.04 | E1 | 2.5 | 35.894074 | 10.598049 | Leisure and fishing boats |
|  |  | E2 | 4.0 | 35.892909 | 10.599694 | Fuel station |
|  |  | E3 | 3.2 | 35.892951 | 10.601285 | Port entrance |
| Heraklion  (Crete, Greece) | 0.87 | H1 | 3.7 | 35.343715 | 25.136632 | Leisure and fishing boats |
|  |  | H3 | 19.5 | 35.346000 | 25.145361 | Passenger ships |
|  |  | H4 | 10.5 | 35.343667 | 25.149000 | Cargo ships |
|  |  | H5 | 19.0 | 35.345083 | 25.152333 | Shipyard |
