# Supplementary material for: Impacts of Anthropogenic Pollutants on Benthic Prokaryotic Communities in Mediterranean Touristic Ports
Source: Front Microbiol. 2020 Jun 9;11:1234. doi: 10.3389/fmicb.2020.01234 (PMC7326019; doi:10.3389/fmicb.2020.01234)
Supplement: Supplementary file 4 [file Table_2.docx]

**Table S2.** Diversity (richness as number of observed OTU, H′: Shannon with an e log base) and evenness (J′: Pielou’s) indices of bacterial and archaeal communities in surface sediments of the ports of Cagliari (C), El Kantaoui (E), and Heraklion (H).

|  | **Bacteria** | | |  | **Archaea** | | |
| --- | --- | --- | --- | --- | --- | --- | --- |
| **Sample** | **Richness** | **H’** | **J’** |  | **Richness** | **H’** | **J’** |
| C1 | 1,899 | 7.3885 | 0.9787 |  | 648 | 6.3182 | 0.9760 |
| C2 | 1,963 | 7.4205 | 0.9787 |  | 617 | 6.2709 | 0.9760 |
| C3 | 1,961 | 7.4310 | 0.9802 |  | 643 | 6.3181 | 0.9771 |
| C4 | 1,912 | 7.3968 | 0.9789 |  | 594 | 6.2291 | 0.9753 |
| C5 | 1,910 | 7.4028 | 0.9799 |  | 602 | 6.2526 | 0.9769 |
| E1 | 1,809 | 7.3282 | 0.9770 |  | 578 | 6.2037 | 0.9755 |
| E2 | 1,686 | 7.2611 | 0.9773 |  | 651 | 6.3137 | 0.9746 |
| E3 | 1,966 | 7.4266 | 0.9793 |  | 582 | 6.1908 | 0.9724 |
| H1 | 1,830 | 7.3542 | 0.9790 |  | 561 | 6.1672 | 0.9743 |
| H3 | 1,814 | 7.3612 | 0.9811 |  | 513 | 6.0850 | 0.9751 |
| H4 | 1,931 | 7.4070 | 0.9790 |  | 578 | 6.1797 | 0.9717 |
| H5 | 654 | 6.2584 | 0.9653 |  | 361 | 5.7166 | 0.9707 |
